# Pineal region collision tumor

**DOI:** 10.1093/jscr/rjaf432

**Published:** 2025-06-18

**Authors:** Petar Marčinković, Andrea Blažević, Danko Muller, Martina Šturlić, Marina Raguž, Darko Orešković, Darko Chudy, Tonko Marinović

**Affiliations:** Department of Neurosurgery, University Hospital Dubrava, Avenija Gojka Šuška 6, 10000 Zagreb, Croatia; Department of Neurosurgery, University Hospital Dubrava, Avenija Gojka Šuška 6, 10000 Zagreb, Croatia; Department of Pathology and Cytology, University Hospital Dubrava, Avenija Gojka Šuška 6, 10000 Zagreb, Croatia; School of Medicine, University of Zagreb, Šalata 2, 10000 Zagreb, Croatia; Department of Diagnostic and Interventional Radiology, University Hospital Dubrava, Avenija Gojka Šuška 6, 10000 Zagreb, Croatia; Department of Neurosurgery, University Hospital Dubrava, Avenija Gojka Šuška 6, 10000 Zagreb, Croatia; School of Medicine, Catholic University of Croatia, Ilica 244, 10000 Zagreb, Croatia; Department of Neurosurgery, University Hospital Dubrava, Avenija Gojka Šuška 6, 10000 Zagreb, Croatia; Department of Neurosurgery, University Hospital Dubrava, Avenija Gojka Šuška 6, 10000 Zagreb, Croatia; School of Medicine, University of Zagreb, Šalata 2, 10000 Zagreb, Croatia; Department of Neurosurgery, University Hospital Dubrava, Avenija Gojka Šuška 6, 10000 Zagreb, Croatia; Chair of Medicine of Sports and Exercise, Faculty of Kinesiology, University of Zagreb, Horvaćanski zavoj 15, 10000 Zagreb, Croatia

**Keywords:** collision tumor, pineocytoma, pilocytic astrocytoma

## Abstract

Collision tumors are exceptionally rare neoplasms characterized by simultaneous presence of two histologically distinct primary brain tumors in the same anatomic location. Their composition can pose a significant therapeutic challenge. Herein, we present a case of a successfully resected pineal region collision tumor composed of a pineocytoma and a pilocytic astrocytoma (PA). A 19-year-old female was admitted with obstructive hydrocephalus due to a pineal gland cyst and underwent surgical excision. Histopathological analysis confirmed a collision tumor comprising pineocytoma and PA. Collision tumors composed of both pineocytoma and PA are extremely rare, with limited cases reported. Their histological architecture presents a diagnostic challenge, requiring meticulous evaluation even by experienced neuropathologists. Given the rarity of such cases, increased awareness of these tumors will facilitate the development of standardized diagnostic criteria and treatment protocols. Multidisciplinary collaboration is essential for optimizing therapeutic strategies tailored to the specific histological composition of collision tumors.

## Introduction

The simultaneous occurrence of multiple CNS (central nervous system) tumors arising from different cell types, or better put, of various histological origins, with no underlying genetic condition nor prior radiation, has seldom been reported in the literature. This extreme condition includes the coexistence of two histologically different primary brain tumors (different glial tumors or glial tumors with meningiomas, adenomas, or other tumors), located in distinct, noncontiguous regions/parts of the brain, with the most common combination being meningioma associated with glioma [1–4]. The incidence of this condition is 0.3% of all brain tumors [5]. Collision tumors present a separate and exceptionally rare entity characterized by the coexistence of two histologically different primary brain neoplasms in the same anatomic location [6–9]. These collision tumors, without a history of radiotherapy or genetic conditions (phakomatosis), may present a therapeutic dilemma in case of significant differences in the treatment of the various components of the collision tumor. We present a case of a 19-year-old female patient presenting with obstructive hydrocephalus due to a pineal region tumor consisting of pilocytic astrocytoma (PA) and pineocytoma as its components, which was successfully surgically treated using a supracerebellar infratentorial approach (SCIT).

## Case report

**Figure 1:**
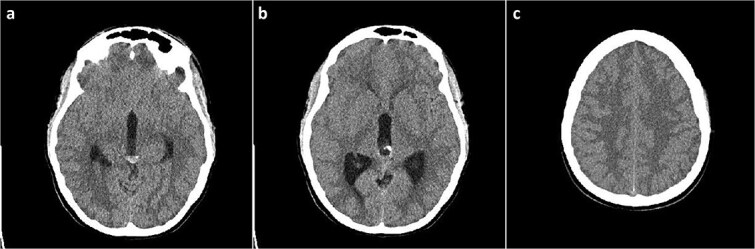
Head CT scan, axial slices. (a) Cystic lesion inside the posterior part of the third ventricle with a hyperdense content in the posterior part of the cyst; (b) probable calcifications in the cyst walls; (c) diffusely obliterated sulci of the convexity as a sign of obstructive hydrocephalus.

A 19-year-old female patient, with a history of a previously neuroradiologically monitored pineal cyst, presented with complaints of a medically refractory headache with nausea and vomiting. A head computed tomography (CT) scan verified a cystic lesion of the pineal gland with marginal calcifications and hyperdense content in the dorsal part with signs of obstructive hydrocephalus (Fig. 1).

**Figure 2:**
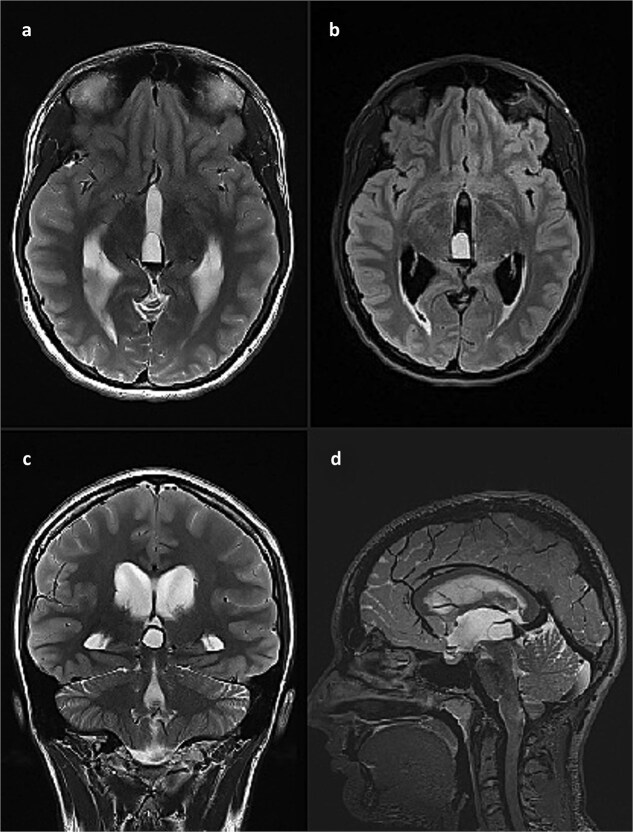
Brain MRI scan. (a) T2 weighted image, axial slice, pineal cyst occupying posterior part of the third ventricle with hemosiderin deposits in the posterior part of the cyst; (b) FLAIR image, axial slice, showing different contents inside the cyst when compared to CSF; (c) T2 weighted image, coronal slice, pineal cyst inside the lower part of the third ventricle, beneath internal cerebral veins; (d) T2 weighted image (SPACE), sagittal slice, showing pineal cyst in front of a rather large vein of Galeni, and compressing the cranial part of the cerebral aqueduct.

This finding was in concordance with a brain magnetic resonance imaging (MRI) done the previous day in another hospital which described hemosiderin deposits within the dorsal part of the cyst, with complete interruption of the cerebrospinal fluid (CSF) communication at the level of mesencephalic aqueduct, and a 4 mm long cerebral tonsil protrusion (Fig. 2).

**Figure 3:**
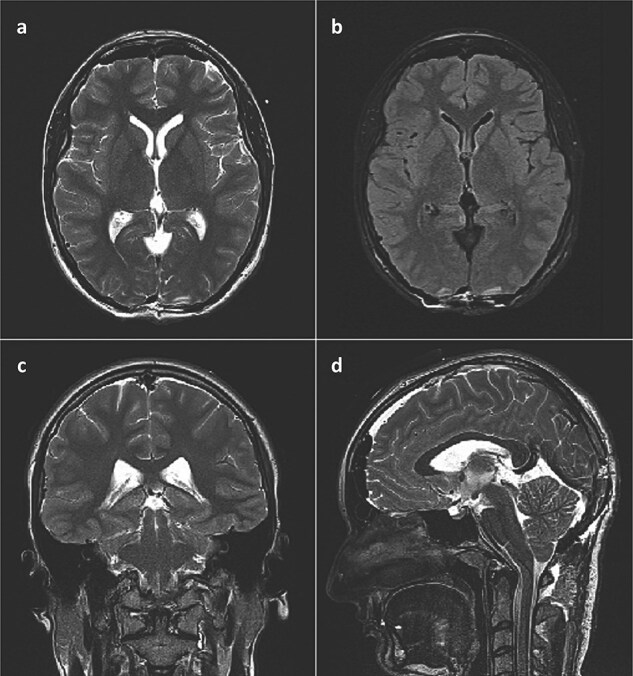
Postoperative brain MRI scan showing no residual tumor. (a) T2 weighted image, axial slice; (b) FLAIR image, axial slice; (c) T2 weighted image, coronal slice; (d) T2 weighted image (SPACE), sagittal slice).

She was admitted to the hospital and an external ventricular drainage was placed in the right lateral ventricle for the treatment of hydrocephalus. The procedure was uneventful and, at that time, ameliorated her symptoms. Five days later, the patient was subjected to a surgical excision of the pineal gland cyst. She was operated on in a sitting position with her neck slightly flexed and head fixed rigidly with the Mayfield head clamp, using a SCIT approach in its standard fashion. Her postoperative recovery was uneventful, with the postoperative head CT scan and brain MRI showing no signs of hemorrhage or tumor residues (Fig. 3).

**Figure 4:**
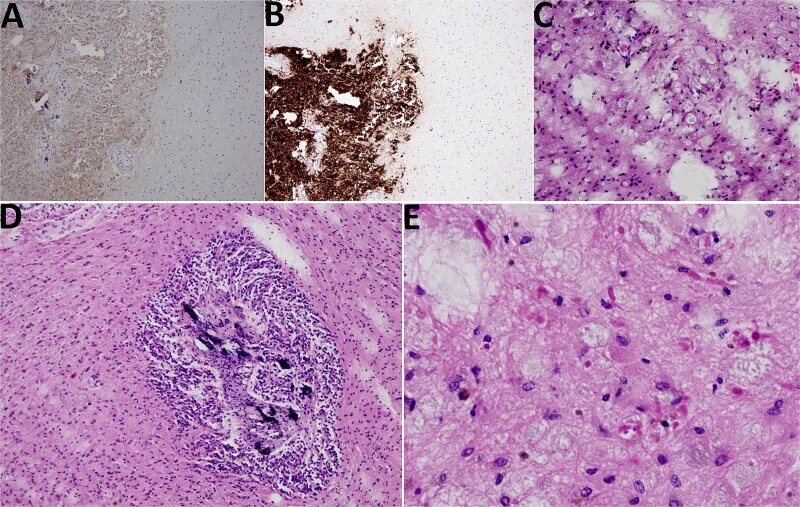
Histological findings. (A) NSE 100× magnification. NSE positivity in pineocytoma (on the left side) in contrast to PA (on the right side). (B) Synaptophysin 100× magnification. Synaptophysin positivity in pineocytoma (lower left quadrant) in contrast to PA (upper left and right side). (C) HE 200× magnification, PA. PA with microcystic vacuoles and Rosenthal fibers. (D) HE 100×. PA surrounding pineocytoma (microcalcifications inside pineocytoma can be seen). (E) HE 400×, PA. PA with Rosenthal fibers.

Histological analysis revealed a tumor tissue composed of lobulated structures made of round to oval cells, without visible mitotic activity, and with visible small calcifications seen focally. The described tumor tissue was surrounded by tissue made of loose clusters of astroglial type of cells with numerous Rosenthal fibers, eosinophilic granular bodies, and glial cells with multiple nuclei. Blood vessels in the mentioned area showed focally mild hyalinization with small extravasates of erythrocytes and siderophage accumulations, while focal small calcifications were also found in the same area. The area of the loose tumor tissue also contained microcystic degeneration with myxoid content. Immunohistochemically, the cellular lobulated areas were synaptophysin and NSE positive, while the loose part of the tumor showed GFAP and OLIG2 positivity. The proliferation index measured by Ki67 was up to 1% in both components of the tumor. In conclusion, histological findings were consistent with a tumor made of two components, pineocytoma (lobulated, more cellular component) and PA (a loose, cystically altered component of the tumor), both classified as WHO grade I (Fig. 4).

The patient was discharged from the hospital with no neurological deficits, after her ventricular drainage was removed. Eight days later, she was admitted due to a CSF leak at the level of the right Kocher’s point where her ventricular drainage was placed previously. No symptoms nor signs of meningitis were observed. She was managed with the placement of ventricular drainage again at the same site and antibiotics prophylactically until her wound completely healed, after which she was discharged from the hospital.

## Discussion

Collision tumors are rare lesions characterized by the coexistence of two histologically distinct primary brain tumors in the same location or in close proximity, necessitating differentiation from tumor-to-tumor metastasis [10]. Unlike metastases, they involve two independent neoplasms arising adjacently, which can complicate diagnosis and treatment [6–9]. While combinations such as meningiomas and gliomas are more common, the occurrence of both pineocytoma and PA is exceptionally rare [1–4].

PA is a slow-growing, well-circumscribed, low-grade glioma commonly found in pediatric patients, with an incidence of 0.84 to 0.91 per 100 000 [11–13]. It typically arises in the cerebellum, optic pathway, brainstem, and spinal cord, and it appears on MRI as a T2 hyperintense, T1 hypointense lesion with a cystic component and an enhancing mural nodule [11]. Pineocytomas are rare, benign pineal tumors, comprising less than 0.4%–1% of all intracranial neoplasms [12, 14, 15], often mimicking pineal cysts, though internal enhancement helps differentiate them [16]. PA symptoms vary by location, while pineocytomas often present with hydrocephalus. Gross total resection is treatment of choice for both; however, pineocytomas may require adjuvant therapy if resection poses high risk [14], and chemotherapy may be used in younger PA patients to avoid the long-term effects of radiotherapy [13].

PA is a low- to moderate-cellular tumor with protoplasmic astrocyte-like cells, Rosenthal fibers, and microcystic changes, while significant mitotic activity may indicate other gliomas [11]. Pineocytomas are composed of well-differentiated pinealocyte-like cells forming pineocytomatous rosettes with strong synaptophysin and neurofilament staining [17]. In this case, histopathology confirmed both tumors, with synaptophysin and NSE positivity in the pineocytoma component and GFAP and OLIG2 positivity in the PA component; both showed a low proliferation index (Ki67 < 1%) [17]. These findings reflect known tumor features but underscore the diagnostic complexity when distinct neoplasms coexist.

Several theories explain collision tumor pathogenesis, including independent tumorigenesis and microenvironmental changes promoting adjacent tumor growth [10]. In this case, the induction theory seems most plausible, suggesting that the primary tumor may have triggered genetic or epigenetic changes in nearby brain tissue, leading to the second neoplasm.

While this case provides some valuable insights, limitations include its retrospective nature, the inability to establish a causal relationship between the two tumors, and the challenge of generalizing findings from a single case. As with all case reports, there is a risk of over-interpretation. Future multicenter studies with larger cohorts could provide deeper insights into the pathophysiology and optimal management strategies for collision tumors. Given the complexity, a multidisciplinary approach remains essential for accurate diagnosis, individualized treatment planning, and optimal patient outcomes.
